# Inflammation and Premature Ageing in Chronic Kidney Disease

**DOI:** 10.3390/toxins12040227

**Published:** 2020-04-04

**Authors:** Thomas Ebert, Sven-Christian Pawelzik, Anna Witasp, Samsul Arefin, Sam Hobson, Karolina Kublickiene, Paul G. Shiels, Magnus Bäck, Peter Stenvinkel

**Affiliations:** 1 Karolinska Institutet, Department of Clinical Science, Intervention and Technology, Division of Renal Medicine, SE-141 86 Stockholm, Sweden; anna.witasp@ki.se (A.W.); samsul.arefin@ki.se (S.A.); sam.hobson@ki.se (S.H.); karolina.kublickiene@ki.se (K.K.); 2 Karolinska Institutet, Department of Medicine Solna, Cardiovascular Medicine Unit, SE-171 76 Stockholm, Sweden; sven-christian.pawelzik@ki.se (S.-C.P.); magnus.back@ki.se (M.B.); 3 Karolinska University Hospital, Theme Heart and Vessels, Division of Valvular and Coronary Disease, SE-171 76 Stockholm, Sweden; 4 University of Glasgow, Wolfson Wohl Cancer Research Centre, College of Medical, Veterinary & Life Sciences, Institute of Cancer Sciences, Glasgow G61 1QH, UK; Paul.Shiels@glasgow.ac.uk

**Keywords:** ageing, chronic kidney disease, end-stage kidney disease, inflammation, premature ageing, senescence, uremic toxins

## Abstract

Persistent low-grade inflammation and premature ageing are hallmarks of the uremic phenotype and contribute to impaired health status, reduced quality of life, and premature mortality in chronic kidney disease (CKD). Because there is a huge global burden of disease due to CKD, treatment strategies targeting inflammation and premature ageing in CKD are of particular interest. Several distinct features of the uremic phenotype may represent potential treatment options to attenuate the risk of progression and poor outcome in CKD. The nuclear factor erythroid 2-related factor 2 (NRF2)–kelch-like erythroid cell-derived protein with CNC homology [ECH]-associated protein 1 (KEAP1) signaling pathway, the endocrine phosphate-fibroblast growth factor-23–klotho axis, increased cellular senescence, and impaired mitochondrial biogenesis are currently the most promising candidates, and different pharmaceutical compounds are already under evaluation. If studies in humans show beneficial effects, carefully phenotyped patients with CKD can benefit from them.

## 1. Introduction—CKD, Inflammation, and Premature Ageing

Chronic kidney disease (CKD) is a major global health burden that contributes to increased morbidity and mortality in affected patients [1]. Inflammation is a key risk factor for CKD progression [2], and recent data from the CANTOS trial suggest that anti-inflammatory treatment in patients with CKD reduces major adverse cardiovascular events [3]. Compared to the general population, patients with CKD also have a highly accelerated ageing process that is characterized by vascular disease; a persistent, low-grade inflammatory status; sarcopenia; and other maladies [4]. Both inflammation and ageing (i.e., “inflammageing”) are established risk factors for mortality in a cluster of “burden of lifestyle diseases”, such as CKD [5], which has been recognized as one of the prototype diseases for premature ageing [6]. Persistent inflammation, premature ageing, and CKD share common regulatory patterns of distinct biological pathways. For instance, the transcription factor nuclear factor erythroid 2-related factor 2 (NRF2) is downregulated in all three conditions [7,8,9]. Thus, both inflammation and premature ageing are major contributing factors to health status and outcome in patients with CKD.

The aim of this review is to summarize the clinical phenotypes of inflammation and premature ageing in CKD. We also summarize the relationship between these two phenotypes. Furthermore, we provide an overview of novel factors contributing to the uremic phenotype, and we describe potential novel targets for the systemic treatment of these interrelated disorders.

## 2. Inflammation in CKD

### 2.1. Uremic Inflammation

An impaired renal function leads to the accumulation of nitrogenous substances in the blood that would normally be excreted in the urine. At progressively increasing concentration, these substances exert toxic effects, which eventually become apparent as symptoms of *uremia*. The altering effects of the uremic milieu on the immune system has been described as *uremic inflammation* [10,11], and include mechanisms of both immunoactivation and immunosuppression. Uremic inflammation resembles the premature ageing phenotype in many ways. On the one hand, it is characterized by an abnormal activation of the innate immune system, especially monocytes [6,12]. This immunoactivation contributes to systemic inflammation via increased synthesis of pro-inflammatory cytokines, such as interleukin (IL)-1, IL-6, and tumor necrosis factor (TNF) [12], and is similar to the chronic low-grade state of systemic inflammation that is associated with an ageing immune system and has been coined “inflammageing” [13]. Furthermore, inflammation is also a major component of other diseases that are independent risk factors for CKD, such as obesity [14]. Importantly, an increased synthesis of pro-inflammatory cytokines and chemokines by senescent cells is one of the main features of cellular senescence [15,16], and has been coined senescence-associated secretory phenotype (SASP). The SASP suggests a further bi-directional link between inflammation and ageing in CKD. On the other hand, a downregulation and reduced function of the adaptive immune system, particularly of T and B lymphocytes, during uremic inflammation parallels “immunosenescence” in the premature ageing phenotype [6,10,12].

The causes of uremic inflammation are multifactorial. Exogenous factors, such as catheterization, exposition to microbial contaminants, or biocompatibility issues during dialysis treatment [10] may play an obvious role in the activation of the immune system and are avoidable using good clinical practice. Possible exposure to bacterial endotoxin, which activates the immune system and contributes to systemic inflammation, can furthermore result from comorbidities, such as gingivitis and periodontitis [17]. Patients with CKD may also show signs of intestinal dysbiosis and increased gut permeability [10], which lead to the presence of bacterial DNA and elevated endotoxin levels, as well as elevated plasma levels of the macrophage-derived cluster of differentiation (CD)14 [10], a co-receptor in the recognition of bacterial endotoxin [18].

Conversely, endogenous factors that provoke uremic inflammation in CKD are linked to metabolic deviations from normal physiology and can be categorized as (i) changes in the mineral metabolism, especially in the levels of phosphate and sodium concentrations; (ii) regulation of oxidative stress; and (iii) increased nonenzymatic glycation. However, these categories are interconnected and may influence each other.

**Figure 1:**
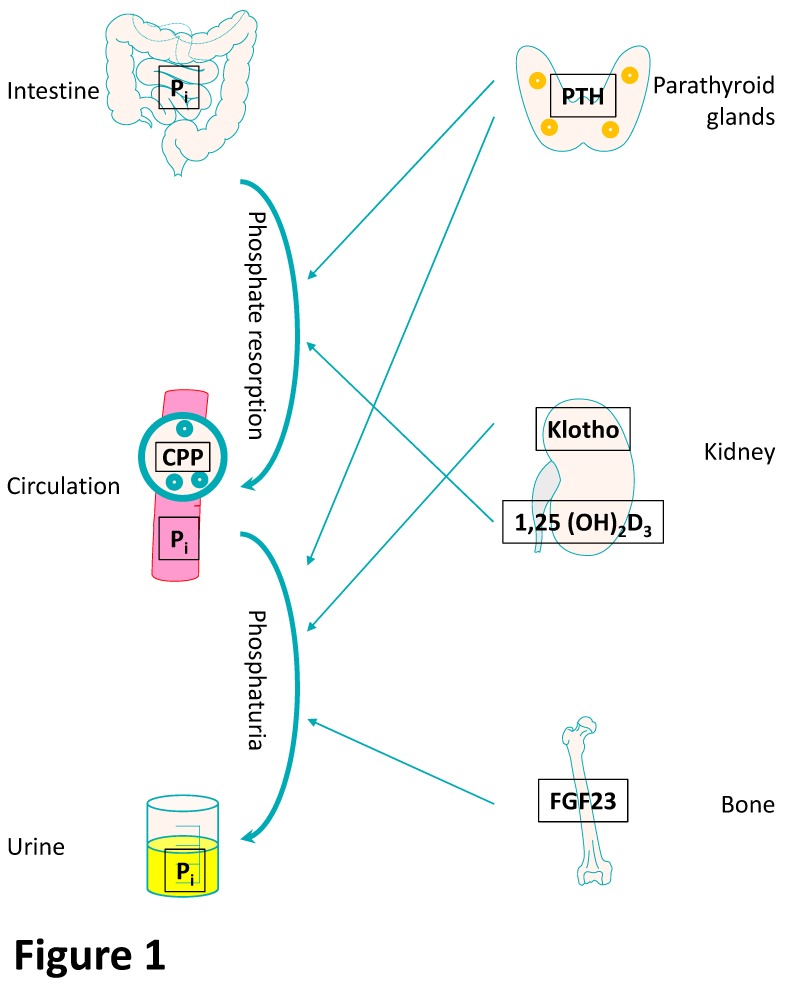
The phosphate-fibroblast growth factor-23 (FGF-23)–klotho endocrine axis. Main effectors of phosphate homeostasis with potential effects on ageing components (simplified overview). Phosphate (P_i_) is taken up by the intestine and accumulates in the circulation of patients with advanced chronic kidney disease (CKD). In the circulation, fetuin A (blue circles)-bound calcium P_i_ in calciprotein particles (CPP) prevents precipitation of calcium P_i_ in the circulation. Increased P_i_ is further regulated by parathyroid hormone (PTH) secreted from the four parathyroid glands (orange circles) at the back of thyroid gland by increasing intestinal P_i_ resorption but also inducing phosphaturia. The 1,25(OH)_2_ vitamin D_3_ metabolite is activated in the kidneys and increases intestinal P_i_ resorption. Furthermore, bone-secreted fibroblast growth factor-23 (FGF-23) also exerts phosphaturic effects through its mandatory co-receptor klotho in the kidneys.

**Figure 2:**
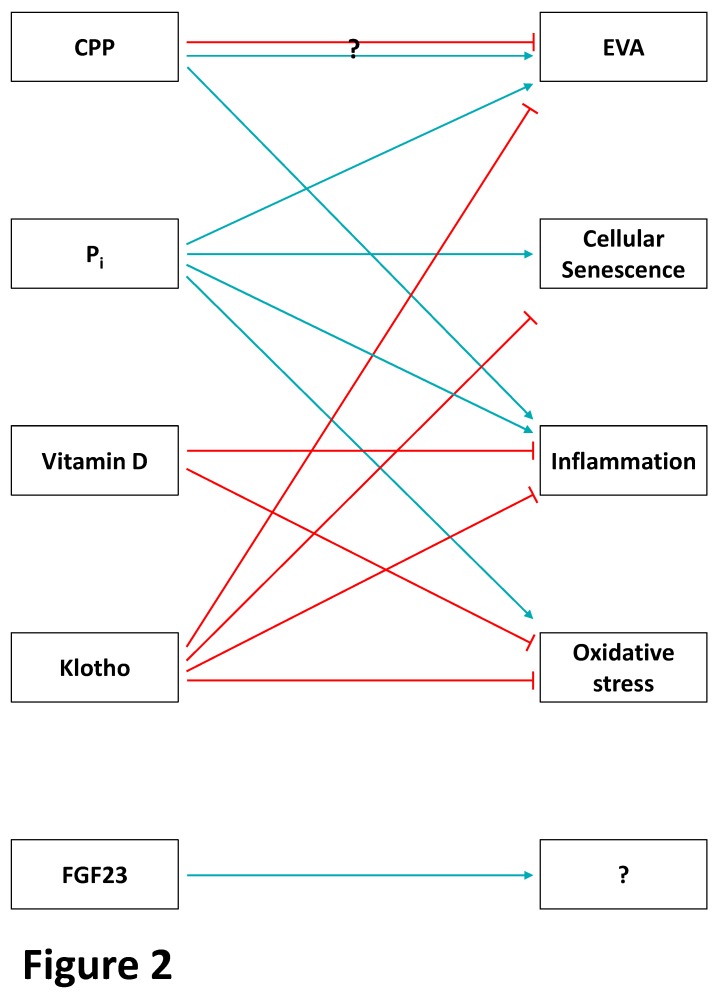
The phosphate-FGF-23–klotho endocrine axis and premature ageing in CKD. Association of the different effectors of the phosphate homeostasis with ageing components (light blue arrows: inducers; red arrows: inhibitors). In advanced CKD, calciprotein particles (CPP) can induce early vascular ageing (EVA) and inflammation. However, contradictory findings also suggest a beneficial role of CPP on EVA. Circulating phosphate (P_i_) promotes EVA, cellular senescence, and oxidative stress by different mechanisms. Vitamin D inhibits inflammation and oxidative stress directly or indirectly. The anti-ageing-associated klotho counteracts EVA, cellular senescence, inflammation, and oxidative stress. Data for fibroblast growth factor-23 (FGF-23) are limited and further studies need to investigate whether FGF-23 directly induces different ageing components or whether the clinical associations to ageing are mediated by other factors, such as P_i_.

The endocrine fibroblast growth factor-23 (FGF-23)–klotho pathway (Figure 1 and Figure 2) is important for the resorption of phosphate in the kidney and is dysregulated in CKD. Decreased renal clearance produces a relative overload of inorganic phosphate (P_i_), which results in hyperphosphatemia and contributes to systemic inflammation and vascular calcification/early vascular ageing (EVA) [19] (Figure 2). Hyperphosphatemia promotes endothelial dysfunction and trans-differentiation of vascular smooth muscle cells (VSMC) into osteoblast-like cells [20]. In bovine aortic smooth muscle cells, high P_i_ promotes an osteogenic phenotype via an inflammatory mechanism involving nuclear factor kappa-light-chain-enhancer of activated B cells (NF-κB) signaling, which increases the generation of reactive oxygen species (ROS). This phenotype can be prevented with the P_i_ binder lanthanum carbonate [21]. Similarly, another P_i_ binder, sevelamer, increases levels of fetuin A, an inhibitor of extracellular matrix mineralization, in patients with CKD [22]. As a negative acute phase protein, low levels of fetuin A indicate systemic inflammation and may shorten telomeres in leukocytes [23]. Furthermore, high P_i_ induces the expression of the pro-inflammatory transcription factor NF-κB in human aortic VSMC [24].

Although P_i_ levels increase in the circulation of CKD patients, sodium accumulates in the tissue under uremic conditions and may lead to chronic systemic inflammation via activation of the p38 mitogen-activated protein kinase (MAPK) pathway and induction of IL-17-producing CD4^+^ T helper cells [6,25].

The transcription factor NRF2 plays an important and ancient role in the anti-oxidant response. Following oxidative stress, NRF2 dissociates from its repressor kelch-like erythroid cell-derived protein with CNC homology [ECH]-associated protein 1 (KEAP1) in the cytosol and subsequently translocates to the nucleus, where it binds to the anti-oxidant response element of numerous promoter regions of over 300 genes encoding anti-oxidant and detoxifying molecules [26]. In peripheral blood mononuclear cells from CKD patients, NRF2 is down-regulated, whereas NF-κB is in turn up-regulated [27]. NF-κB plays a key role in regulating the inflammatory response and is stimulated by ROS [10]. This suggests that uremia induces an impaired NRF2 system in CKD and hemodialysis (HD) patients, which contributes to the pathogenesis of oxidative stress and inflammation [27]. Importantly, increased oxidative stress and its sequalae are major contributors to premature atherosclerosis and calcification [28], resulting in increased cardiovascular (CV) morbidity and mortality in CKD [29,30]. Retained uremic toxins may further both become substrates for oxidative injury and increase the burden of oxidative stress [29,30].

Advanced glycation end-products (AGE) are formed from reducing sugars and biomolecules such as proteins, lipids, and nucleic acids by a sequence of nonenzymatic glycation reactions, collectively known as the Maillard reaction [26,31]. Exogenous sources of AGE originate from diet [32] and tobacco smoking [33]. Endogenously, AGE formation is promoted by hyperglycemia, but also by high levels of oxidative stress [26]. In fact, oxidative stress and AGE formation mutually stimulate each other [34]. AGE increase the levels of ROS via activation of Nicotinamide adenine dinucleotide phosphate (NADPH) oxidase through interaction with the advanced glycation end product-specific receptor (RAGE), as well as through receptor-independent pathways, whereas ROS decrease the levels of glyoxalase I (Glo-1), an enzyme that detoxifies AGE precursor molecules [26]. In CKD, AGE accumulate as a result from both a decreased renal clearance and increased formation [26]. AGE–RAGE interaction activates the NF-κB pathway and thus perpetuates uremic inflammation via release of pro-inflammatory cytokines, such as IL-1, IL-6, and TNF [35].

### 2.2. Consequences of Inflammation on Premature Ageing in CKD

Persistent uremic inflammation directly promotes premature ageing and further increases inflammatory effects through a vicious circle. For instance, NF-κB promotes cellular senescence and accelerated ageing but it is also activated in senescent cells [36,37,38]. In more detail, senescent cells promote inflammation through the transcription factor GATA4, which increases NF-κB to initiate the SASP [39]. Furthermore, cell replication is amplified in acute and chronic inflammation, resulting in increased telomere attrition, which is directly linked to cellular ageing [40]. Importantly, the association between telomere length and inflammation also seems to be reciprocally. Thus, mice deficient in the telomerase genes telomerase RNA component (*TERC*) and telomerase reverse transcriptase *(TERT)* show a higher amount of mRNA expression of various pro-inflammatory cytokines [41]. In a cross-sectional study, the negative association of leukocyte telomere length with circulating inflammatory markers was also confirmed in prevalent HD patients [23]. Moreover, increased oxidative stress in CKD and adverse lifestyle factors (e.g., smoking and diet), as well as psychological stress, may further promote telomere shortening through inflammation [40].

## 3. Premature Ageing in CKD

Because the kidneys are involved in the regulation of many systemic processes, patients with CKD are at a high risk to develop multiple systemic complications, including rheologic, metabolic, immunologic, CV, and other disturbances [42]. Importantly, several of these complications show similarities with the ageing process, such as CV diseases (CVD), sarcopenia, bone disease, as well as frailty [43,44], cognitive dysfunction [44], immune deficiency [12], and finally mortality [1]. Thus, patients with CKD appear to have a highly accelerated ageing process as compared to healthy subjects or non-CKD patients. We present possible causes for the premature ageing process in CKD, focusing on uremic toxins including AGE, the endocrine phosphate-FGF-23–klotho axis, as well as NRF2 as a master regulator of mitochondrial dysfunction/oxidative stress. Importantly, all pathways are linked to hallmarks of the ageing process [45].

### 3.1. Uremic Toxins and Premature Ageing in CKD

When renal function declines during the progression of CKD, distinct organic compounds accumulate [46], and signs and symptoms of *uremia* occur. The European Uremic Toxins (EUTox) Work Group has provided a comprehensive overview of circulating solutes in CKD [47] describing a large number of uremic toxins with significantly differing circulating levels in end stage kidney disease (ESKD), which can be sorted into three groups: (1) free water-soluble low-molecular-weight solutes, (2) protein-bound solutes, and (3) middle molecules [47]. For a variety of these uremic toxins, associations with the ageing process have been described. Thus, the protein-bound uremic toxins indoxyl sulfate and p-cresyl sulfate induce oxidative stress by different mechanisms [48]. Using an in vitro approach, indoxyl sulfate attenuates mitochondrial activity in human renal proximal tubule epithelial cells [49] and human umbilical vein endothelial cells [50], thereby affecting the cellular metabolic capacity. Furthermore, indoxyl sulfate induces markers of senescence, including senescence-associated beta-galactosidase (SA-β-gal), through oxidative stress [51]. Indoxyl sulfate and p-cresyl sulfate also increased mitochondrial autophagy (“mitophagy”) [52]. Although autophagy in general has been suggested as a compensatory and pro-survival mechanism [53], it could also have adverse effects when mitochondrial mass decreased at a level beyond cellular compensation [52]. Thus, mitochondrial dysfunction is one of the key mechanisms linking uremic toxins with hallmarks of the ageing process [45] through increased oxidative stress [54]. It should be noted that indoxyl sulfate dose-dependently decreases protein levels of klotho in human aortic VSMC in vitro by inducing DNA methylation of the klotho-coding *Kl* gene [55]. In accordance, patients with ESKD have increased promoter hypermethylation of the *Kl* gene [55]. Conversely, the inhibition of uremic toxins should have beneficial effects on ageing markers. As an example drug, the oral sorbent AST-120 adsorbs uremic toxins and their precursors within the gastrointestinal tract [56]. AST-120 dose-dependently decreases circulating indoxyl sulfate [56] and was, therefore, tested in several clinical trials [56]. Results of these trials were heterogenous depending on the prespecified primary endpoints, follow-up period, as well as baseline characteristics of the included subjects. In conclusion, there was no effect of adding AST-120 to standard therapy in patients with moderate to severe CKD on a primary composite renal end point in the EPPIC trials [57]. It should be noted, however, that on the basis of the results of a subgroup analysis in the EPPIC trials, AST-120 delayed the time of reaching the primary renal end point in patients from the United States [58].

AGE are another group of uremic toxins that accumulate in CKD [26]. Besides the well-known associations of AGE levels and CVD (mostly investigated in patients with diabetes mellitus) [59,60], AGE and AGE-detoxifying enzymes including Glo-1 also have direct effects on ageing processes. Thus, the AGE–RAGE axis induces renal cytosolic oxidative stress and mitochondrial dysfunction, as well as inflammation [26,61]. The AGE–RAGE axis further promotes premature senescence in proximal tubular epithelial cells via endoplasmatic reticulum stress and p21 activation in an in vivo and in vitro approach [62]. Moreover, AGE-induced endoplasmatic reticulum stress also increases p16 protein expression in tubular epithelial cells in vitro in a dose- and time-dependent manner through the activating transcription factor 4 [63]. Conversely, reduced renal senescence in tubule iss observed in rodents with a transgenic overexpression of the AGE-detoxifying enzyme Glo-1 in vivo and in vitro [54]. More recently, AGE have been shown to induce renal senescence in mesangial cells via the RAGE/Signal transducer and activator of transcription 5 (STAT5) pathway and inhibiting autophagy [64].

**Figure 3:**
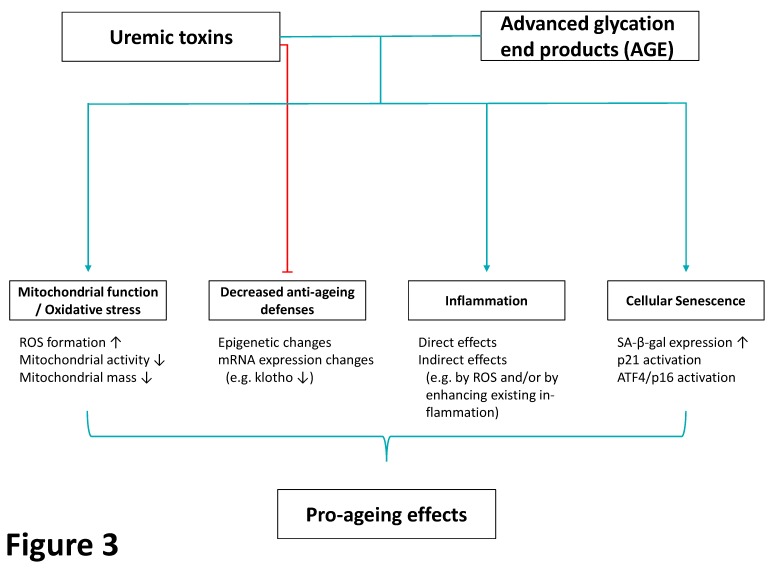
Mechanisms by which uremic toxins and advanced glycation end products (AGE) induce pro-ageing effects. Both uremic toxins and AGE have adverse effects on mitochondrial function including formation of reactive oxygen species (ROS) but also structural disturbances. They impair anti-ageing defenses of the organism, and induce inflammation, as well as cellular senescence. All effects result in an adverse, pro-ageing milieu caused by uremic toxins and AGE. Light blue arrows: inducers; red arrows: inhibitors. ATF4: activating transcription factor 4, ROS: reactive oxygen species, SA-β-gal: senescence-associated beta-galactosidase.

Taken together, uremic toxins, including AGE, have distinct and adverse effects on the pro- and anti-oxidative milieu, mitochondrial function, inflammation, and finally cellular senescence; all of which are hallmarks of the ageing process [45] (Figure 3).

### 3.2. The Endocrine Phosphate-FGF-23–Klotho Axis and Premature Ageing in CKD

A clear negative association of serum P_i_ levels with life span has been reported in mammals [8]. Phosphate homeostasis is regulated by a distinct endocrine network involving vitamin D, parathyroid hormone (PTH), klotho, and FGF-23 (Figure 1). In more detail, P_i_ originating from dietary intake is absorbed by the small intestine by an active transcellular transport or a paracellular pathway [65]. In hypophosphatemia, either normal or increased levels of the active metabolite 1,25(OH)_2_ vitamin D_3_ are observed in humans [66], and 1,25(OH)_2_ vitamin D_3_ increases the rate of intestinal P_i_ resorption [67]. Furthermore, the parathyroid glands are affected by elevated serum P_i_ levels and release PTH, which enhances urinary P_i_ excretion by PTH-induced removal of the renal sodium P_i_ cotransporters from the apical membrane [68]. Bone-derived FGF-23 limits hyperphosphatemia by inducing phosphaturia and suppressing vitamin D secretion [69]. FGF-23 signals through endocrine FGF receptor complexes [70] and also associates with distinct metabolic components [71]. FGF-23 requires klotho protein as a co-receptor [70]. The full length klotho protein, which is predominantly expressed in the proximal and distal tubules of the kidneys [72], is cleaved and circulates as a soluble klotho fragment serving as a surrogate marker of renal klotho expression [70]. In the circulation, calciprotein particles (CPP) “capture” calcium (Ca) and P_i_ with the help of the adipokine fetuin A and prevent the precipitation of Ca-P_i_ [70,73].

Phosphate induces EVA via different mechanisms, and higher serum P_i_ concentrations, even if they are within the normal range, have been found to be associated with microvascular dysfunction in a Dutch, population-based cohort study [74] (Figure 2). In human aortic VSMC, P_i_ treatment arrests the cell cycle [75], mediating cellular senescence. In vitro, P_i_ further induces vascular calcification and production of ROS [76]. Mediated by Ca-P_i_ crystals and a disturbed microRNA homeostasis, P_i_ has additional indirect and negative effects on EVA [77]. Importantly, these in vitro data on EVA are supported by a Scottish general population study showing that P_i_ is linked to markers of biological age, that is, reduced telomere length, DNA methylation content, and chronological age [78].

Adverse effects of P_i_ on EVA are at least in part mediated by the Wnt/beta-catenin pathway [79,80]. Thus, high P_i_ activates the Wnt/beta-catenin pathway in VSMC in vitro, promoting VSMC calcification and osteogenic transition [79]. Conversely, shRNA-mediated knockdown of beta-catenin in CKD rats on a high-phosphate diet reduced vascular calcification [79]. Interestingly, klotho is an inhibitor of the Wnt/beta-catenin pathway [70], ameliorating high P_i_-induced VSMC calcification [81], as well as inhibiting osteogenic transition of these cells [82,83]. Taken together, P_i_ induces EVA at least in part by the Wnt/beta-catenin pathway, and different treatment approaches potentially mitigate the adverse effects of P_i_ on EVA, including klotho.

Besides preventing the precipitation of Ca-P_i_ (Figure 2), CPP can induce pro-inflammatory effects in VSMC in vitro [84], further linking inflammation and EVA in CKD. Furthermore, CPP differ between healthy subjects and patients with ESKD and can, therefore, promote widespread calcification in CKD [85].

Because of the above-mentioned evidence of a direct association between P_i_ and premature ageing in CKD, non-pharmaceutical prevention strategies could potentially reduce the burden of disease, including changes in lifestyle. However, there is a lack of studies linking diets that lower P_i_ levels to a beneficial clinical outcome [86,87]. Furthermore, whereas low-P_i_ diets can reduce FGF-23 levels in CKD [88], there was no dose-dependent effect of P_i_ lowering on FGF-23 reduction when low- and very low phosphate diets were compared in a recent short-term, randomized, crossover trial in 35 ESKD patients [89]. Therefore, non-pharmacological lifestyle-changing prevention strategies might provide a benefit on components of the ageing process, but randomized controlled trials are needed to prove these associations.

Vitamin D has also been linked to distinct components of the ageing process (Figure 2). The negative acute phase reactant vitamin D [90] mitigates oxidative stress at least in part through increased expression of anti-oxidant regulators, including NRF2 and klotho [91,92,93]. Thus, calcitriol increased renal and circulating klotho expression, whereas it reduced markers of oxidative stress in uninephrectomized, spontaneously hypertensive rats [94]. These data have been confirmed in a recent randomized controlled trial showing increased circulating klotho and total antioxidant capacity after 12 weeks of cholecalciferol treatment compared to the placebo group [95]. Human ex vivo studies indicate that vitamin D exerts anti-inflammatory effects on uremic lymphocytes under renal replacement therapy (RRT) [96]. Meta-analyses demonstrating reduced circulating markers of inflammation in mostly non-CKD subjects on vitamin D supplementation [97,98], further supporting a significant role of vitamin D on the hallmarks of ageing. However, these associations were not seen in CKD patients on vitamin D supplementation [99]. Furthermore, two systematic reviews and meta-analyses did not find strong evidence for a beneficial effect of vitamin D supplementation on arterial stiffness [100] and endothelial function [101], two major components of EVA. Thus, future studies need to validate whether vitamin D has direct effects on premature ageing in CKD or whether its effects are mediated by NRF2, klotho, and others.

FGF-23 has been associated with an adverse outcome in CKD in large studies [102]. However, only a few experimental studies have investigated causal effects of FGF-23 on inflammation and premature ageing as hallmarks in the pathogenesis of CKD and its complications. Thus, FGF-23 induces hepatic inflammation in CKD [103]. Furthermore, some [104,105] but not all [106] studies suggest that FGF-23 might induce EVA. Taken together, data for direct FGF-23 effects on *renal* inflammation and premature ageing in CKD are limited (Figure 2).

Simic et al. [107] recently showed that kidney-derived glycerol-3-phosphate in the renal vein correlated with circulating FGF-23 levels, and they further demonstrated a novel kidney–bone axis by which renal glycerol-3-phosphate increases bone-secreted FGF-23. Future studies need to investigate whether renal glycerol-3-phosphate could also be a potential target for preventing premature ageing.

With respect to premature ageing, klotho (Figure 2) is clearly the most interesting member of this endocrine axis, as Kuro-o et al. [70] described a syndrome that resembles human ageing in mice that have a defect in the klotho-coding *kl* gene. When renal function declines, klotho levels decrease in humans and in rodents with CKD [70]. Klotho-overexpressing mice with CKD have an increased phosphaturia, as well as an improved renal function and EVA phenotype [72]. In mice with an ICR-derived glomerulonephritis, overexpression of the *kl* gene reduces blood urea nitrogen levels, proteinuria, renal SA-β-gal activity, and the development of glomerular and tubulointerstitial changes [108]. Mechanistically, klotho-overexpressing mice show an improved mitochondrial function, as well as a reduced mitochondrial DNA damage and oxidative stress, in the kidneys [108].

Interestingly, both high P_i_ and inflammation decrease renal *Kl* mRNA expression in vitro [109]. Thus, 5/6 nephrectomized rats on a 0.4% phosphorus diet showed reduced renal klotho protein expression compared to sham-operated animals [109]. Lipopolysaccharide-induced inflammation further reduced klotho expression in all groups [109]. In vivo and ex vivo pharmacological inhibition of NF-κB and Wnt recovered reduced klotho expression after lipopolysaccharide treatment [109] further suggesting that inflammation and the Wnt/beta-catenin pathway are crucially involved in the downregulation of renal klotho in CKD. The pro-fibrotic transforming growth factor (TGF)-β, which is upregulated in CKD, is another mediator of reduced renal klotho in CKD, facilitating adverse Wnt/beta-catenin activation in the kidney [110].

Besides these in vivo data in renal tissue, klotho exerts beneficial effects on the endothelium including attenuation of inflammatory and adhesion molecules, as well as augmented NO production and vasorelaxation [111]. Moreover, klotho-deficient mice show increased vascular calcification [112,113], further supporting a beneficial role of klotho on EVA. In addition, the above-described role of uremic toxins in reducing klotho expression further strengthens these effects in CKD. Finally and most importantly, because overexpression of klotho in mice extends their lifespan [70], klotho is one of the most promising anti-ageing targets in CKD.

### 3.3. Mitochondrial Dysfunction/Oxidative Stress and Premature Ageing in CKD

Patients with CKD show increased oxidative stress on the basis of an over-production of ROS, as well as decreased anti-oxidant defenses [29,114]. In general, ROS are generated from the reduction of oxygen and can induce oxidation of important macromolecules, including proteins, lipids, carbohydrates, and DNA [29,114], thereby exerting toxic effects on mitochondria [115]. Thus, sufficient anti-oxidative mechanisms are necessary to counteract the excessive ROS formation in cells. One of the most interesting anti-oxidative targets interfering with ageing is the transcription factor NRF2, which is a key regulator of anti-oxidative enzymes. When oxidative stress occurs, NRF2 translocates to the nucleus and activates >300 distinct genes that have an anti-oxidant response element in their promoter regions [26,116]. Mitochondrial dysfunction in CKD is associated with low NRF2 expression in human skeletal muscle [117]. NRF2 is also negatively associated with different other age-related lifestyle diseases [9] and rare progeroid syndromes [7]. Thus, patients with Hutchinson–Gilford progeria syndrome (HGPS) show an impaired activity of the NRF2 pathway, and reactivation of NRF2 in ex vivo cells of patients with HGPS can reverse ageing defects [118]. Mechanistically, NRF2 knockdown in wildtype fibroblasts increases oxidative stress and recapitulates cellular senescence defects of HGPS [118]. Furthermore, caveolin-induced NRF2 inhibition induces senescence in murine adipocytes in vitro and, conversely, less inhibition of NRF2-dependent anti-oxidant signaling results in decreased senescence, as assessed by SA-β-gal and p21^Waf1/Cip1^ [119]. Molecular markers of vascular senescence, that is, p16*^INK4a^* and p21^Waf1/Cip1^, as well as cytokines of the SASP, are increased in NRF2-deficient mice compared to control mice [120]. Klotho also exerts some of its beneficial effects on EVA by activating the NRF2 pathway [121]. Furthermore, because testosterone ameliorates age-related renal fibrosis in mice via activation of NRF2 signaling [122], the effects of sex hormones and their supplementation in CKD also need attention. In ESKD, reduced NRF2 expression in peripheral blood mononuclear cells has been reported in addition to an up-regulation of pro-inflammatory NF-κB [27]. Furthermore, uremic toxins correlate positively with NF-κB expression and negatively with NRF2 expression in peripheral blood mononuclear cells [123]. Accordingly, rats undergoing 5/6 nephrectomy also show an impaired activation of NRF2 in the kidneys [124]. Conversely, the NRF2 agonist bardoxolone reduces tubular cell mitochondrial damage and improves redox balance and mitochondrial function in a CKD mouse model [125]. Taken together, mitochondrial dysfunction and oxidative stress promote premature ageing. Because the protective NRF2 pathway is attenuated in CKD and other burden of lifestyle diseases associated with ageing, this offers the potential for future NRF2 agonist-based treatment.

## 4. Secondary Premature Ageing by CKD-Causing Diseases and CKD Treatment

As described above, many facets of CKD contribute to premature ageing. However, distinct causes of CKD contribute to premature ageing, irrespective of renal function, and are summarized below.

### 4.1. Glomerular Diseases Excluding Diabetic Kidney Disease (DKD)

In contrast to other CKD causes, the incidence of IgA nephropathy (IgAN) does not increase with age and is, therefore, higher in children and young adults as compared to elderly subjects [126,127]. In Asian cohorts of IgAN, renal biopsies show higher expressions of p16*^INK4^* [128], p21 [129], and reduced klotho [128] compared to controls. Furthermore, p16*^INK4^*, as well as klotho, correlated with IgAN-related renal fibrosis [128]. Because other glomerular diseases, including focal segmental glomerulosclerosis and minimal change disease, are also associated with an increased renal expression of the senescence marker p16^INK4A^ [127], glomerular diseases seem to have direct relations to premature ageing. Patients with Fabry disease have shorter leukocyte telomere length compared to controls [130]. Thus, this rare renal disease could also serve as a model of premature ageing [131].

### 4.2. Polycystic Kidney Disease as a Model of Adverse Anti-Senescence

Patients with autosomal dominant polycystic kidney disease (ADPKD) often progress to CKD and ESKD [132]. Interestingly and in contrast to most other underlying CKD causes, the senescence marker p21^Waf1/Cip1^ is decreased in both human and rodent ADPKD, suggesting that an anti-senescent milieu promotes cyst formation [127]. In accordance with this hypothesis, the cyclin-dependent kinase inhibitor roscovitine reduces cyst formation in mice with PKD [127]. In contrast, nephronophthisis, another cystic kidney disease, is characterized by cellular senescence [133]. Furthermore, senolytic treatment in mice with nephronophthisis type 7 improves cystic area, inflammation, and renal fibrosis [134]. These contradictory findings indicate that senescence must be viewed as disease-specific and in the context of a spectrum ranging from beneficial to adverse effects.

### 4.3. Obesity, Diabetes Mellitus, and DKD

Obesity is a major predictor contributing to type 2 diabetes (T2D), CKD, CVD, and increased mortality [135,136]. Besides being risk factors for CKD, obesity and T2D also contribute to cellular senescence per se [137,138]. Furthermore, the adverse effects of an increased fat mass are at least partly related to the secretion of proteins from adipocytes into the circulation, that is, adipocytokines. Indeed, adipocytokines associate with adverse metabolic status and the SASP [71,139]. Interestingly, treatment of obese mice with the senolytic agents dasatinib and quercetin improves glucose tolerance, enhances insulin sensitivity, lowers circulating inflammatory mediators, and increases levels of the beneficial adipocytokine adiponectin [140]. Furthermore, distinct adipocytokines can also contribute to attenuated vascular calcification in CKD, such as chemerin [141].

With respect to DKD, both humans and mice with DKD have an increased expression of senescence markers, including SA-β-gal, p16^INK4A^, and p21^Waf1/Cip1^, in different renal cell types, and diabetic, p21-deficient mice are protected from DKD compared to diabetic wildtype mice [127]. However, future studies need to validate whether hyperglycemia rather than DKD itself might cause this pro-senescent phenotype.

### 4.4. CKD-/ESKD Treatment-Associated Ageing (Dialysis, Transplantation)

As the uremic milieu associates with premature ageing and senescence, one could speculate that RRT (dialysis and renal transplantation [Rtx]) improves a prematurely aged phenotype. However, the dialysis procedure itself exerts pro-inflammatory and pro-oxidative effects due to multiple factors, such as bio-incompatibility of dialysis membranes or fluids, contaminated/polluted dialysis water, intravenous iron treatment, activation of the renin–angiotensin–aldosterone system (RAAS), and depletion of anti-oxidants [6] potentially resulting in adverse effects on ageing processes in RRT. Ageing-associated phenotypes are also observed after Rtx. Thus, patients undergoing Rtx have an increased risk for complications, including ischemia-reperfusion injury (IRI) and allograft rejection during and after Rtx. These complications can result in an accelerated senescence as assessed by p21^Waf1/Cip1^ and p16^Ink4a^, as well as telomere shortening [127]. Furthermore, transplant biopsies show strong p16^INK4a^ staining beyond the amount predicted by chronological age [142]. Conversely, short-term inhibition of the senescence promoter p53 reduces IRI-induced senescence and improves kidney outcome in mice [143]. Immunosuppressive treatment of the recipients can further result in therapy-induced senescent cells that remain in the transplanted kidney and mediate adverse pro-ageing signals [144]. Thus, patients receiving mycophenolate mofetil after Rtx have an increased telomere attrition compared to azathioprine-treated patients, supporting a direct effect of immunosuppressive treatment on ageing [145]. Taken together, RRT by either dialysis or Rtx does not arrest ageing processes, and RRT might even accelerate the ageing phenotype.

## 5. Approaches for Handling of Inflammation and Premature Ageing in CKD

Inflammation and premature ageing are hallmarks in the pathogenesis of CKD and its many complications, having highly detrimental effects on health status, quality of life, and mortality. However, current treatment recommendations indicate that each single risk factor for CKD progression and its complications must be treated separately and, at the final stage, RRT has to be provided [42]. This approach led to substantial improvements in reducing the mortality of patients with CKD. For example, in patients with T1D, the use of novel insulins, new glucose monitoring devices, and the widespread usage of RAAS blockers has improved the outcome [146,147]. However, because the global burden of disease due to CKD is increasing [148], systemic approaches for the treatment of CKD and its complications are warranted to target the underlying hallmarks of CKD, that is, inflammation and premature ageing. Some of these are summarized below.

### 5.1. NRF2–KEAP1 Signaling Pathway

Several groups have independently shown beneficial associations and effects of the NRF2 system as a key regulator of anti-oxidative enzymes in CKD, and promote NRF2 as a multiorgan protector.

Concluding the findings discussed above, treatment of the repressed NRF2 system in CKD can improve oxidative stress and mitochondrial function [125,149], inflammation [27,123,149], as well as premature ageing [122], in particular EVA [120,121].

NRF2-inducing treatment options include nutritional components, exercise [150], and pharmaceutical compounds targeting NRF2 or inhibiting the binding of NRF2 to KEAP1 [116]. Although the above-mentioned beneficial effects of NRF2 have drawn interest to this molecular “health promoter”, potential caveats should be acknowledged. Firstly, NRF2 has dual roles in its association with carcinogenesis, as well as cancer progression and therapy. Thus, NRF2 activation in normal cells can prevent cancer initiation [151]. In contrast, prolonged NRF2 activation is involved in cancer promotion, progression, and treatment resistance [151]. Consequently, each NRF2-based approach must carefully investigate oncogenic risk as a potential side effect. Secondly, despite promising initial results, previous treatment approaches for patients with DKD using the NRF2 agonist bardoxolone have been stopped due to an excess of heart failure hospitalizations among those assigned to bardoxolone [7]. However, because patients with risk factors for heart failure could be identified and excluded, bardoxolone is currently being re-investigated in different clinical trials comprising patients with CKD and underlying pathologies [116]. When patients in these studies are carefully selected, the NRF2 agonist might have beneficial effects on several hallmarks of the uremic phenotype, such as inflammation, oxidative stress, as well as on premature ageing. Thus, NRF2 is currently a promising and the clinically most advanced signaling pathway for the treatment of both inflammation and premature ageing in CKD. It should be kept in mind that it is also possible to target NRF2 by nutraceuticals, such as sulforaphane [7].

### 5.2. Klotho Pathway

Kidney-derived klotho is of particular interest as a potential treatment for inflammation and premature ageing in CKD, because klotho-deficient mice and patients with CKD have similar phenotypes, such as EVA and a pro-inflammatory status, and klotho is related to ageing in both humans and mice [70,72]. Similar to the NRF2 system, klotho is repressed in CKD, and stimulation of klotho can ameliorate oxidative stress and mitochondrial function [108], renal fibrosis [152], inflammation [72], as well as premature ageing [108], including EVA [55,81,82,83,111,112,113].

Importantly, and possibly in contrast to the NRF2–KEAP1 signaling pathway, klotho is down-regulated in several cancers and is also recognized as a potential anti-tumor therapy [153].

Therapeutic approaches to stimulate klotho expression can aim for the reactivation of endogenous klotho or the administration of exogenous klotho [72]. Several drugs increase endogenous klotho [72]. For instance, the thiazolidinedione pioglitazone protects against renal injury in ageing by an increased expression of klotho [72]. Furthermore, the vitamin D analog paricalcitol induces the tissue-dependent expression of klotho in the kidneys and increases serum and urinary klotho levels in rodent CKD models [72]. In vitro data using murine internal medulla collecting duct epithelial cells further suggest that statins induce *klotho* mRNA expression [72]. Moreover, because angiotensin II and aldosterone decrease *klotho* mRNA expression in vitro and in vivo [70], RAAS blockers potentially reverse the decrease in *klotho* expression in rodents [70]. Recently, testosterone was positively correlated with circulating klotho levels in both men and women, and the association sustained significance after adjustment for cortisol and markers of renal function but not chronological age [154]. However, and in contrast to this study, a randomized controlled trial of transdermal testosterone does not find changes in soluble klotho levels between the verum and placebo group [155]. Finally, direct administration of exogenous, soluble klotho has also been proven effective for increasing circulating klotho levels, as well as protecting against acute kidney injury and CKD [72,156]. It has to be pointed out that, except for direct klotho administration, each of the mentioned pharmacological compounds is already approved for the use in CKD, and for some but not all of these, positive renal outcome data in humans with CKD are available [157]. However, the relative contribution of increased klotho on the outcome of these human studies has not been analyzed thus far. Hence, the direct effect of klotho on uremic inflammation and premature ageing needs to be addressed. However, preliminary safety data appear to be more attractive compared to targeting the NRF2–KEAP1 signaling pathway.

## 6. Conclusions

Persistent low-grade inflammation and premature ageing are hallmarks of the uremic phenotype and contribute to impaired health status, reduced quality of life, and premature mortality. Several potential treatment options targeting distinct features of the uremic phenotype may attenuate the risk of progression and poor outcome. Because the burden of disease due to CKD is huge, systemic treatment approaches targeting the underlying hallmarks of CKD, that is, inflammation and premature ageing, are currently being investigated. The NRF2–KEAP1 signaling pathway, the endocrine klotho axis, increased cellular senescence, and impaired mitochondrial biogenesis are currently the most promising candidates, and different pharmaceutical compounds are already in evaluation. If randomized controlled trials show beneficial effects, patients with distinct CKD phenotypes can benefit from them.

## References

[1] Bikbov B., Purcell C.A., Levey A.S., Smith M., Abdoli A., Abebe M., Adebayo O.M., Afarideh M., Agarwal S.K., Agudelo-Botero M. (2020). Global, regional, and national burden of chronic kidney disease, 1990–2017: A systematic analysis for the global burden of disease study 2017. Lancet.

[2] Ruiz-Ortega M., Rayego-Mateos S., Lamas S., Ortiz A., Rodrigues-Diez R.R. (2020). Targeting the progression of chronic kidney disease. Nat. Rev. Nephrol..

[3] Ridker P.M., MacFadyen J.G., Glynn R.J., Koenig W., Libby P., Everett B.M., Lefkowitz M., Thuren T., Cornel J.H. (2018). Inhibition of interleukin-1β by canakinumab and cardiovascular outcomes in patients with chronic kidney disease. J. Am. Coll. Cardiol..

[4] Dai L., Qureshi A.R., Witasp A., Lindholm B., Stenvinkel P. (2019). Early vascular ageing and cellular senescence in chronic kidney disease. Comput. Struct. Biotechnol. J..

[5] Hobson S., Arefin S., Kublickiene K., Shiels P.G., Stenvinkel P. (2019). Senescent cells in early vascular ageing and bone disease of chronic kidney disease—A novel target for treatment. Toxins.

[6] Kooman J.P., Kotanko P., Schols A.M.W.J., Shiels P.G., Stenvinkel P. (2014). Chronic kidney disease and premature ageing. Nat. Rev. Nephrol..

[7] Stenvinkel P., Meyer C.J., Block G.A., Chertow G.M., Shiels P.G. (2019). Understanding the role of the cytoprotective transcription factor nuclear factor erythroid 2–related factor 2—Lessons from evolution, the animal kingdom and rare progeroid syndromes. Nephrol. Dial. Transplant..

[8] Stenvinkel P., Painer J., Kuro-o M., Lanaspa M., Arnold W., Ruf T., Shiels P.G., Johnson R.J. (2018). Novel treatment strategies for chronic kidney disease: Insights from the animal kingdom. Nat. Rev. Nephrol..

[9] Cuadrado A., Manda G., Hassan A., Alcaraz M.J., Barbas C., Daiber A., Ghezzi P., León R., López M.G., Oliva B. (2018). Transcription factor NRF2 as a therapeutic target for chronic diseases: A systems medicine approach. Pharmacol. Rev..

[10] Kooman J.P., Dekker M.J., Usvyat L.A., Kotanko P., van der Sande F.M., Schalkwijk C.G., Shiels P.G., Stenvinkel P. (2017). Inflammation and premature aging in advanced chronic kidney disease. Am. J. Physiol. Ren. Physiol..

[11] Cobo G., Lindholm B., Stenvinkel P. (2018). Chronic inflammation in end-stage renal disease and dialysis. Nephrol. Dial. Transplant..

[12] Sato Y., Yanagita M. (2019). Immunology of the ageing kidney. Nat. Rev. Nephrol..

[13] Franceschi C., Garagnani P., Parini P., Giuliani C., Santoro A. (2018). Inflammaging: A new immune—Metabolic viewpoint for age-related diseases. Nat. Rev. Endocrinol..

[14] Pawelzik S.-C., Avignon A., Idborg H., Boegner C., Stanke-Labesque F., Jakobsson P.-J., Sultan A., Bäck M. (2019). Urinary prostaglandin D2 and E2 metabolites associate with abdominal obesity, glucose metabolism, and triglycerides in obese subjects. Prostaglandins Other Lipid Mediat..

[15] Ferenbach D.A., Bonventre J.V. (2015). Mechanisms of maladaptive repair after AKI leading to accelerated kidney ageing and CKD. Nat. Rev. Nephrol..

[16] Kirkland J.L., Tchkonia T. (2017). Cellular senescence: A translational perspective. EBioMedicine.

[17] Hickey N.A., Shalamanova L., Whitehead K.A., Dempsey-Hibbert N., van der Gast C., Taylor R.L. (2020). Exploring the putative interactions between chronic kidney disease and chronic periodontitis. Crit. Rev. Microbiol..

[18] Van Maldeghem I., Nusman C.M., Visser D.H. (2019). Soluble CD14 subtype (sCD14-ST) as biomarker in neonatal early-onset sepsis and late-onset sepsis: A systematic review and meta-analysis. BMC Immunol..

[19] Carracedo M., Artiach G., Witasp A., Clària J., Carlström M., Laguna-Fernandez A., Stenvinkel P., Bäck M. (2019). The G-protein coupled receptor ChemR23 determines smooth muscle cell phenotypic switching to enhance high phosphate-induced vascular calcification. Cardiovasc. Res..

[20] Abbasian N., Burton J.O., Herbert K.E., Tregunna B.-E., Brown J.R., Ghaderi-Najafabadi M., Brunskill N.J., Goodall A.H., Bevington A. (2015). Hyperphosphatemia, phosphoprotein phosphatases, and microparticle release in vascular endothelial cells. J. Am. Soc. Nephrol..

[21] Zhao W.-H., Gou B.-D., Zhang T.-L., Wang K. (2012). Lanthanum chloride bidirectionally influences calcification in bovine vascular smooth muscle cells. J. Cell. Biochem..

[22] Zhou Z., Ji Y., Ju H., Chen H., Sun M. (2019). Circulating fetuin-A and risk of all-cause mortality in patients with chronic kidney disease: A systematic review and meta-analysis. Front. Physiol..

[23] Carrero J.J., Stenvinkel P., Fellström B., Qureshi A.R., Lamb K., Heimbürger O., Bárány P., Radhakrishnan K., Lindholm B., Soveri I. (2008). Telomere attrition is associated with inflammation, low fetuin-A levels and high mortality in prevalent haemodialysis patients. J. Intern. Med..

[24] Voelkl J., Tuffaha R., Luong T.T.D., Zickler D., Masyout J., Feger M., Verheyen N., Blaschke F., Kuro-o M., Tomaschitz A. (2018). Zinc inhibits phosphate-induced vascular calcification through TNFAIP3-Mediated suppression of NF-κB. J. Am. Soc. Nephrol..

[25] Mikolajczyk T.P., Guzik T.J. (2019). Adaptive immunity in hypertension. Curr. Hypertens. Rep..

[26] Stinghen A.E.M., Massy Z.A., Vlassara H., Striker G.E., Boullier A. (2016). Uremic toxicity of advanced glycation end products in CKD. J. Am. Soc. Nephrol..

[27] Pedruzzi L.M., Cardozo L.F.M.F., Daleprane J.B., Stockler-Pinto M.B., Monteiro E.B., Leite M., Vaziri N.D., Mafra D. (2015). Systemic inflammation and oxidative stress in hemodialysis patients are associated with down-regulation of Nrf2. J. Nephrol..

[28] Mercier N., Pawelzik S.-C., Pirault J., Carracedo M., Persson O., Wollensack B., Franco-Cereceda A., Bäck M. (2020). Semicarbazide-Sensitive Amine Oxidase Increases in Calcific Aortic Valve Stenosis and Contributes to Valvular Interstitial Cell Calcification. Oxid. Med. Cell. Longev..

[29] Daenen K., Andries A., Mekahli D., Van Schepdael A., Jouret F., Bammens B. (2019). Oxidative stress in chronic kidney disease. Pediatr. Nephrol..

[30] Huang M., Zheng L., Xu H., Tang D., Lin L., Zhang J., Li C., Wang W., Yuan Q., Tao L. (2020). Oxidative stress contributes to vascular calcification in patients with chronic kidney disease. J. Mol. Cell. Cardiol..

[31] Rabbani N., Thornalley P.J. (2018). Advanced glycation end products in the pathogenesis of chronic kidney disease. Kidney Int..

[32] Snelson M., Coughlan M.T. (2019). Dietary advanced glycation end products: Digestion, metabolism and modulation of gut microbial ecology. Nutrients.

[33] Chapman S., Mick M., Hall P., Mejia C., Sue S., Wase B.A., Nguyen M.A., Whisenant E.C., Wilcox S.H., Winden D. (2018). Cigarette smoke extract induces oral squamous cell carcinoma cell invasion in a receptor for advanced glycation end-products-dependent manner. Eur. J. Oral Sci..

[34] Nowotny K., Jung T., Höhn A., Weber D., Grune T. (2015). Advanced glycation end products and oxidative stress in type 2 diabetes mellitus. Biomolecules.

[35] Sanajou D., Ghorbani Haghjo A., Argani H., Aslani S. (2018). Age-Rage axis blockade in diabetic nephropathy: Current status and future directions. Eur. J. Pharmacol..

[36] Taniguchi K., Karin M. (2018). NF-κB, inflammation, immunity and cancer: Coming of age. Nat. Rev. Immunol..

[37] Osorio F.G., Soria-Valles C., Santiago-Fernández O., Freije J.M.P., López-Otín C., Jeon K.W., Galluzzi L. (2016). Chapter Four—NF-κB signaling as a driver of ageing. International Review of Cell and Molecular Biology.

[38] Costantino S., Paneni F., Cosentino F. (2016). Ageing, metabolism and cardiovascular disease. J. Physiol..

[39] Kang C., Xu Q., Martin T.D., Li M.Z., Demaria M., Aron L., Lu T., Yankner B.A., Campisi J., Elledge S.J. (2015). The DNA damage response induces inflammation and senescence by inhibiting autophagy of GATA4. Science.

[40] Zhang J., Rane G., Dai X., Shanmugam M.K., Arfuso F., Samy R.P., Lai M.K.P., Kappei D., Kumar A.P., Sethi G. (2016). Ageing and the telomere connection: An intimate relationship with inflammation. Ageing Res. Rev..

[41] Chen R., Zhang K., Chen H., Zhao X., Wang J., Li L., Cong Y., Ju Z., Xu D., Williams B.R.G. (2015). Telomerase deficiency causes alveolar stem cell senescence-associated low-grade inflammation in lungs. J. Biol. Chem..

[42] Romagnani P., Remuzzi G., Glassock R., Levin A., Jager K.J., Tonelli M., Massy Z., Wanner C., Anders H.-J. (2017). Chronic kidney disease. Nat. Rev. Dis. Primer.

[43] Chowdhury R., Peel N.M., Krosch M., Hubbard R.E. (2017). Frailty and chronic kidney disease: A systematic review. Arch. Gerontol. Geriatr..

[44] Drew D.A., Weiner D.E., Sarnak M.J. (2019). Cognitive impairment in CKD: Pathophysiology, management, and prevention. Am. J. Kidney Dis..

[45] López-Otín C., Blasco M.A., Partridge L., Serrano M., Kroemer G. (2013). The hallmarks of aging. Cell.

[46] Underwood C.F., Hildreth C.M., Wyse B.F., Boyd R., Goodchild A.K., Phillips J.K. (2017). Uraemia: An unrecognized driver of central neurohumoral dysfunction in chronic kidney disease?. Acta Physiol..

[47] Duranton F., Cohen G., Smet R.D., Rodriguez M., Jankowski J., Vanholder R., Argiles A., European Uremic Toxin Work Group (2012). Normal and pathologic concentrations of uremic toxins. J. Am. Soc. Nephrol..

[48] Fujii H., Goto S., Fukagawa M. (2018). Role of uremic toxins for kidney, cardiovascular, and bone dysfunction. Toxins.

[49] Mutsaers H.A.M., Wilmer M.J.G., Reijnders D., Jansen J., van den Broek P.H.H., Forkink M., Schepers E., Glorieux G., Vanholder R., van den Heuvel L.P. (2013). Uremic toxins inhibit renal metabolic capacity through interference with glucuronidation and mitochondrial respiration. Biochim. Biophys. Acta BBA Mol. Basis Dis..

[50] Lee W.-C., Li L.-C., Chen J.-B., Chang H.-W. (2015). Indoxyl sulfate-induced oxidative stress, mitochondrial dysfunction, and impaired biogenesis are partly protected by vitamin C and N-Acetylcysteine. Sci. World J..

[51] Muteliefu G., Shimizu H., Enomoto A., Nishijima F., Takahashi M., Niwa T. (2012). Indoxyl sulfate promotes vascular smooth muscle cell senescence with upregulation of p53, p21, and prelamin A through oxidative stress. Am. J. Physiol. Cell Physiol..

[52] Sun C.-Y., Cheng M.-L., Pan H.-C., Lee J.-H., Lee C.-C. (2017). Protein-bound uremic toxins impaired mitochondrial dynamics and functions. Oncotarget.

[53] Carracedo M., Persson O., Saliba-Gustafsson P., Artiach G., Ehrenborg E., Eriksson P., Franco-Cereceda A., Bäck M. (2019). Upregulated autophagy in calcific aortic valve stenosis confers protection of valvular interstitial cells. Int. J. Mol. Sci..

[54] Hirakawa Y., Jao T.-M., Inagi R. (2017). Pathophysiology and therapeutics of premature ageing in chronic kidney disease, with a focus on glycative stress. Clin. Exp. Pharmacol. Physiol..

[55] Chen J., Zhang X., Zhang H., Liu T., Zhang H., Teng J., Ji J., Ding X. (2016). Indoxyl sulfate enhance the hypermethylation of klotho and promote the process of vascular calcification in chronic kidney disease. Int. J. Biol. Sci..

[56] Asai M., Kumakura S., Kikuchi M. (2019). Review of the efficacy of AST-120 (KREMEZIN^®^) on renal function in chronic kidney disease patients. Ren. Fail..

[57] Schulman G., Berl T., Beck G.J., Remuzzi G., Ritz E., Arita K., Kato A., Shimizu M. (2015). Randomized placebo-controlled EPPIC trials of AST-120 in CKD. J. Am. Soc. Nephrol..

[58] Schulman G., Berl T., Beck G.J., Remuzzi G., Ritz E., Shimizu M., Shobu Y., Kikuchi M. (2016). The effects of AST-120 on chronic kidney disease progression in the United States of America: A post hoc subgroup analysis of randomized controlled trials. BMC Nephrol..

[59] Koska J., Saremi A., Howell S., Bahn G., Courten B.D., Ginsberg H., Beisswenger P.J., Reaven P.D. (2018). Advanced glycation end products, oxidation products, and incident cardiovascular events in patients with type 2 diabetes. Diabetes Care.

[60] De Vos L.C., Lefrandt J.D., Dullaart R.P.F., Zeebregts C.J., Smit A.J. (2016). Advanced glycation end products: An emerging biomarker for adverse outcome in patients with peripheral artery disease. Atherosclerosis.

[61] Chaudhuri J., Bains Y., Guha S., Kahn A., Hall D., Bose N., Gugliucci A., Kapahi P. (2018). The role of advanced glycation end products in aging and metabolic diseases: Bridging association and causality. Cell Metab..

[62] Liu J., Huang K., Cai G.-Y., Chen X.-M., Yang J.-R., Lin L.-R., Yang J., Huo B.-G., Zhan J., He Y.-N. (2014). Receptor for advanced glycation end-products promotes premature senescence of proximal tubular epithelial cells via activation of endoplasmic reticulum stress-dependent p21 signaling. Cell Signal..

[63] Liu J., Yang J.-R., Chen X.-M., Cai G.-Y., Lin L.-R., He Y.-N. (2015). Impact of ER stress-regulated ATF4/p16 signaling on the premature senescence of renal tubular epithelial cells in diabetic nephropathy. Am. J. Physiol. Cell Physiol..

[64] Shi M., Yang S., Zhu X., Sun D., Sun D., Jiang X., Zhang C., Wang L. (2019). The RAGE/STAT5/autophagy axis regulates senescence in mesangial cells. Cell Signal..

[65] Knöpfel T., Himmerkus N., Günzel D., Bleich M., Hernando N., Wagner C.A. (2019). Paracellular transport of phosphate along the intestine. Am. J. Physiol. Gastrointest. Liver Physiol..

[66] Florenzano P., Cipriani C., Roszko K.L., Fukumoto S., Collins M.T., Minisola S., Pepe J. (2020). Approach to patients with hypophosphataemia. Lancet Diabetes Endocrinol..

[67] Hernando N., Wagner C.A. (2018). Mechanisms and regulation of intestinal phosphate absorption. Compr. Physiol..

[68] Jacquillet G., Unwin R.J. (2019). Physiological regulation of phosphate by vitamin D, parathyroid hormone (PTH) and phosphate (Pi). Pflügers Arch. Eur. J. Physiol..

[69] Vervloet M. (2019). Renal and extrarenal effects of fibroblast growth factor 23. Nat. Rev. Nephrol..

[70] Kuro-o M. (2019). The klotho proteins in health and disease. Nat. Rev. Nephrol..

[71] Ebert T., Gebhardt C., Scholz M., Wohland T., Schleinitz D., Fasshauer M., Blüher M., Stumvoll M., Kovacs P., Tönjes A. (2018). Relationship between 12 adipocytokines and distinct components of the metabolic syndrome. J. Clin. Endocrinol. Metab..

[72] Zou D., Wu W., He Y., Ma S., Gao J. (2018). The role of klotho in chronic kidney disease. BMC Nephrol..

[73] Bäck M., Aranyi T., Cancela M.L., Carracedo M., Conceição N., Leftheriotis G., Macrae V., Martin L., Nitschke Y., Pasch A. (2019). Endogenous calcification inhibitors in the prevention of vascular calcification: A consensus statement from the COST action EuroSoftCalcNet. Front. Cardiovasc. Med..

[74] Ginsberg C., Houben A.J.H.M., Malhotra R., Berendschot T.T.J.M., Dagnelie P.C., Kooman J.P., Webers C.A., Stehouwer C.D.A., Ix J.H. (2019). Serum phosphate and microvascular function in a population-based cohort. Clin. J. Am. Soc. Nephrol..

[75] Rahabi-Layachi H., Ourouda R., Boullier A., Massy Z.A., Amant C. (2015). Distinct effects of inorganic phosphate on cell cycle and apoptosis in human vascular smooth muscle cells. J. Cell. Physiol..

[76] Zhao M.-M., Xu M.-J., Cai Y., Zhao G., Guan Y., Kong W., Tang C., Wang X. (2011). Mitochondrial reactive oxygen species promote p65 nuclear translocation mediating high-phosphate-induced vascular calcification in vitro and in vivo. Kidney Int..

[77] Vervloet M.G., Sezer S., Massy Z.A., Johansson L., Cozzolino M., Fouque D., ERA–EDTA Working Group on Chronic Kidney Disease–Mineral and Bone Disorders and the European Renal Nutrition Working Group (2017). The role of phosphate in kidney disease. Nat. Rev. Nephrol..

[78] McClelland R., Christensen K., Mohammed S., McGuinness D., Cooney J., Bakshi A., Demou E., MacDonald E., Caslake M., Stenvinkel P. (2017). Accelerated ageing and renal dysfunction links lower socioeconomic status and dietary phosphate intake. Aging.

[79] Yao L., Sun Y., Sun W., Xu T., Ren C., Fan X., Sun L., Liu L., Feng J., Ma J. (2015). High phosphorus level leads to aortic calcification via β-catenin in chronic kidney disease. Am. J. Nephrol..

[80] Cai T., Sun D., Duan Y., Wen P., Dai C., Yang J., He W. (2016). WNT/β-catenin signaling promotes VSMCs to osteogenic transdifferentiation and calcification through directly modulating Runx2 gene expression. Exp. Cell Res..

[81] Chen Y.-X., Huang C., Duan Z.-B., Xu C.-Y., Chen Y. (2019). Klotho/FGF23 axis mediates high phosphate-induced vascular calcification in vascular smooth muscle cells via Wnt7b/β-catenin pathway. Kaohsiung J. Med. Sci..

[82] Chen T., Mao H., Chen C., Wu L., Wang N., Zhao X., Qian J., Xing C. The Role and Mechanism of α-Klotho in the Calcification of Rat Aortic Vascular Smooth Muscle Cells. https://www.hindawi.com/journals/bmri/2015/194362/.

[83] Mencke R., Hillebrands J.-L. (2017). The role of the anti-ageing protein Klotho in vascular physiology and pathophysiology. Ageing Res. Rev..

[84] Aghagolzadeh P., Bachtler M., Bijarnia R., Jackson C., Smith E.R., Odermatt A., Radpour R., Pasch A. (2016). Calcification of vascular smooth muscle cells is induced by secondary calciprotein particles and enhanced by tumor necrosis factor-α. Atherosclerosis.

[85] Viegas C.S.B., Santos L., Macedo A.L., Matos A.A., Silva A.P., Neves P.L., Staes A., Gevaert K., Morais R., Vermeer C. (2018). Chronic kidney disease circulating calciprotein particles and extracellular vesicles promote vascular calcification. Arterioscler. Thromb. Vasc. Biol..

[86] Barreto F.C., Barreto D.V., Massy Z.A., Drüeke T.B. (2019). Strategies for phosphate control in patients with CKD. Kidney Int. Rep..

[87] Elder G.J., Malik A., Lambert K. (2018). Role of dietary phosphate restriction in chronic kidney disease. Nephrology.

[88] Tsai W.-C., Wu H.-Y., Peng Y.-S., Hsu S.-P., Chiu Y.-L., Chen H.-Y., Yang J.-Y., Ko M.-J., Pai M.-F., Tu Y.-K. (2018). Effects of lower versus higher phosphate diets on fibroblast growth factor-23 levels in patients with chronic kidney disease: A systematic review and meta-analysis. Nephrol. Dial. Transplant..

[89] Tsai W.-C., Wu H.-Y., Peng Y.-S., Hsu S.-P., Chiu Y.-L., Yang J.-Y., Chen H.-Y., Pai M.-F., Lin W.-Y., Hung K.-Y. (2019). Short-term effects of very-low-phosphate and low-phosphate diets on fibroblast growth factor 23 in hemodialysis patients: A randomized crossover trial. Clin. J. Am. Soc. Nephrol..

[90] Czarnik T., Czarnik A., Gawda R., Gawor M., Piwoda M., Marszalski M., Maj M., Chrzan O., Said R., Rusek-Skora M. (2018). Vitamin D kinetics in the acute phase of critical illness: A prospective observational study. J. Crit. Care.

[91] Norris K.C., Olabisi O., Barnett M.E., Meng Y.-X., Martins D., Obialo C., Lee J.E., Nicholas S.B. (2018). The role of vitamin D and oxidative stress in chronic kidney disease. Int. J. Environ. Res. Public Health.

[92] Berridge M.J. (2017). Vitamin D deficiency accelerates ageing and age-related diseases: A novel hypothesis. J. Physiol..

[93] Haussler M.R., Whitfield G.K., Haussler C.A., Sabir M.S., Khan Z., Sandoval R., Jurutka P.W., Litwack G. (2016). Chapter eight–1,25-Dihydroxyvitamin D and Klotho: A tale of two renal hormones coming of age. Vitamins & Hormones.

[94] Takenaka T., Inoue T., Ohno Y., Miyazaki T., Nishiyama A., Ishii N., Suzuki H. (2014). Calcitriol supplementation improves endothelium-dependent vasodilation in rat hypertensive renal injury. Kidney Blood Press. Res..

[95] Jebreal Azimzadeh M., Shidfar F., Jazayeri S., Hosseini A.F., Ranjbaran F. (2020). Effect of vitamin D supplementation on klotho protein, antioxidant status and nitric oxide in the elderly: A randomized, double-blinded, placebo-controlled clinical trial. Eur. J. Integr. Med..

[96] Carvalho J.T.G., Schneider M., Cuppari L., Grabulosa C.C., Aoike D.T., Redublo B.M.Q., Batista M.C., Cendoroglo M., Moyses R.M., Dalboni M.A. (2017). Cholecalciferol decreases inflammation and improves vitamin D regulatory enzymes in lymphocytes in the uremic environment: A randomized controlled pilot trial. PLoS ONE.

[97] Mansournia M.A., Ostadmohammadi V., Doosti-Irani A., Ghayour-Mobarhan M., Ferns G., Akbari H., Ghaderi A., Talari H.R., Asemi Z. (2018). The effects of vitamin d supplementation on biomarkers of inflammation and oxidative stress in diabetic patients: A systematic review and meta-analysis of randomized controlled trials. Horm. Metab. Res..

[98] Kruit A., Zanen P. (2016). The association between vitamin D and C-reactive protein levels in patients with inflammatory and non-inflammatory diseases. Clin. Biochem..

[99] Hu C., Wu X. (2019). Effect of vitamin D supplementation on vascular function and inflammation in patients with chronic kidney disease: A controversial issue. Ther. Apher. Dial..

[100] Rodríguez A.J., Scott D., Srikanth V., Ebeling P. (2016). Effect of vitamin D supplementation on measures of arterial stiffness: A systematic review and meta-analysis of randomized controlled trials. Clin. Endocrinol. (Oxf.).

[101] Hussin A.M., Ashor A.W., Schoenmakers I., Hill T., Mathers J.C., Siervo M. (2017). Effects of vitamin D supplementation on endothelial function: A systematic review and meta-analysis of randomised clinical trials. Eur. J. Nutr..

[102] Gutiérrez O.M., Mannstadt M., Isakova T., Rauh-Hain J.A., Tamez H., Shah A., Smith K., Lee H., Thadhani R., Jüppner H. (2008). Fibroblast growth factor 23 and mortality among patients undergoing hemodialysis. N. Engl. J. Med..

[103] Singh S., Grabner A., Yanucil C., Schramm K., Czaya B., Krick S., Czaja M.J., Bartz R., Abraham R., Di Marco G.S. (2016). Fibroblast growth factor 23 directly targets hepatocytes to promote inflammation in chronic kidney disease. Kidney Int..

[104] Silswal N., Touchberry C.D., Daniel D.R., McCarthy D.L., Zhang S., Andresen J., Stubbs J.R., Wacker M.J. (2014). FGF23 directly impairs endothelium-dependent vasorelaxation by increasing superoxide levels and reducing nitric oxide bioavailability. Am. J. Physiol. Endocrinol. Metab..

[105] Verkaik M., Juni R.P., van Loon E.P.M., van Poelgeest E.M., Kwekkeboom R.F.J., Gam Z., Richards W.G., Ter Wee P.M., Hoenderop J.G., Eringa E.C. (2018). FGF23 impairs peripheral microvascular function in renal failure. Am. J. Physiol. Heart Circ. Physiol..

[106] Lindberg K., Olauson H., Amin R., Ponnusamy A., Goetz R., Taylor R.F., Mohammadi M., Canfield A., Kublickiene K., Larsson T.E. (2013). Arterial klotho expression and FGF23 effects on vascular calcification and function. PLoS ONE.

[107] Simic P., Kim W., Zhou W., Pierce K.A., Chang W., Sykes D.B., Aziz N.B., Elmariah S., Ngo D., Pajevic P.D. (2020). Glycerol-3-phosphate is an FGF23 regulator derived from the injured kidney. J. Clin. Investig..

[108] Haruna Y., Kashihara N., Satoh M., Tomita N., Namikoshi T., Sasaki T., Fujimori T., Xie P., Kanwar Y.S. (2007). Amelioration of progressive renal injury by genetic manipulation of Klotho gene. Proc. Natl. Acad. Sci. USA.

[109] Rodríguez-Ortiz M.E., Díaz-Tocados J.M., Muñoz-Castañeda J.R., Herencia C., Pineda C., Martínez-Moreno J.M., Montes de Oca A., López-Baltanás R., Alcalá-Díaz J., Ortiz A. (2020). Inflammation both increases and causes resistance to FGF23 in normal and uremic rats. Clin. Sci..

[110] Zhou L., Li Y., Zhou D., Tan R.J., Liu Y. (2013). Loss of klotho contributes to kidney injury by derepression of Wnt/β-catenin signaling. J. Am. Soc. Nephrol..

[111] Vila Cuenca M., Hordijk P.L., Vervloet M.G. (2019). Most exposed: The endothelium in chronic kidney disease. Nephrol. Dial. Transplant..

[112] Ter Braake A.D., Smit A.E., Bos C., van Herwaarden A.E., Alkema W., van Essen H.W., Bravenboer N., Vervloet M.G., Hoenderop J.G.J., de Baaij J.H.F. (2020). Magnesium prevents vascular calcification in Klotho deficiency. Kidney Int..

[113] Leibrock C.B., Alesutan I., Voelkl J., Pakladok T., Michael D., Schleicher E., Kamyabi-Moghaddam Z., Quintanilla-Martinez L., Kuro-o M., Lang F. (2015). NH4Cl treatment prevents tissue calcification in klotho deficiency. J. Am. Soc. Nephrol..

[114] Ravarotto V., Simioni F., Pagnin E., Davis P.A., Calò L.A. (2018). Oxidative stress—Chronic kidney disease—Cardiovascular disease: A vicious circle. Life Sci..

[115] Bhargava P., Schnellmann R.G. (2017). Mitochondrial energetics in the kidney. Nat. Rev. Nephrol..

[116] Cuadrado A., Rojo A.I., Wells G., Hayes J.D., Cousin S.P., Rumsey W.L., Attucks O.C., Franklin S., Levonen A.-L., Kensler T.W. (2019). Therapeutic targeting of the NRF2 and KEAP1 partnership in chronic diseases. Nat. Rev. Drug Discov..

[117] Liu C., Gidlund E., Witasp A., Qureshi A.R., Söderberg M., Thorell A., Nader G.A., Barany P., Stenvinkel P., von Walden F. (2019). Reduced skeletal muscle expression of mitochondrial derived peptides humanin and MOTS-C and Nrf2 in chronic kidney disease. Am. J. Physiol.-Ren. Physiol..

[118] Kubben N., Zhang W., Wang L., Voss T.C., Yang J., Qu J., Liu G.-H., Misteli T. (2016). Repression of the antioxidant NRF2 pathway in premature aging. Cell.

[119] Volonte D., Liu Z., Musille P.M., Stoppani E., Wakabayashi N., Di Y.-P., Lisanti M.P., Kensler T.W., Galbiati F. (2013). Inhibition of nuclear factor-erythroid 2–related factor (Nrf2) by caveolin-1 promotes stress-induced premature senescence. Mol. Biol. Cell.

[120] Fulop G.A., Kiss T., Tarantini S., Balasubramanian P., Yabluchanskiy A., Farkas E., Bari F., Ungvari Z., Csiszar A. (2018). Nrf2 deficiency in aged mice exacerbates cellular senescence promoting cerebrovascular inflammation. GeroScience.

[121] Romero A., Hipólito-Luengo Á.S., Villalobos L.A., Vallejo S., Valencia I., Michalska P., Pajuelo-Lozano N., Sánchez-Pérez I., León R., Bartha J.L. (2019). The angiotensin-(1-7)/Mas receptor axis protects from endothelial cell senescence via klotho and Nrf2 activation. Aging Cell.

[122] Zhang G., Kang Y., Zhou C., Cui R., Jia M., Hu S., Ji X., Yuan J., Cui H., Shi G. (2018). Amelioratory effects of testosterone propionate on age-related renal fibrosis via suppression of TGF-β1/Smad signaling and activation of Nrf2-ARE signaling. Sci. Rep..

[123] Stockler-Pinto M.B., Soulage C.O., Borges N.A., Cardozo L.F.M.F., Dolenga C.J., Nakao L.S., Pecoits-Filho R., Fouque D., Mafra D. (2018). From bench to the hemodialysis clinic: Protein-bound uremic toxins modulate NF-κB/Nrf2 expression. Int. Urol. Nephrol..

[124] Zhang H., Wang J., Wang Y., Gao C., Gu Y., Huang J., Wang J., Zhang Z. (2019). Salvianolic acid a protects the kidney against oxidative stress by activating the Akt/GSK-3 *β*/Nrf2 signaling pathway and inhibiting the NF- *κ* B signaling pathway in 5/6 nephrectomized rats. Oxid. Med. Cell. Longev..

[125] Nagasu H., Sogawa Y., Kidokoro K., Itano S., Yamamoto T., Satoh M., Sasaki T., Suzuki T., Yamamoto M., Wigley W.C. (2019). Bardoxolone methyl analog attenuates proteinuria-induced tubular damage by modulating mitochondrial function. FASEB J..

[126] Lai K.N., Tang S.C.W., Schena F.P., Novak J., Tomino Y., Fogo A.B., Glassock R.J. (2016). IgA nephropathy. Nat. Rev. Dis. Primer.

[127] Sturmlechner I., Durik M., Sieben C.J., Baker D.J., van Deursen J.M. (2017). Cellular senescence in renal ageing and disease. Nat. Rev. Nephrol..

[128] Yamada K., Doi S., Nakashima A., Kawaoka K., Ueno T., Doi T., Yokoyama Y., Arihiro K., Kohno N., Masaki T. (2015). Expression of age-related factors during the development of renal damage in patients with IgA nephropathy. Clin. Exp. Nephrol..

[129] Jiang H., Liang L., Qin J., Lu Y., Li B., Wang Y., Lin C., Zhou Q., Feng S., Yip S.H. (2016). Functional networks of aging markers in the glomeruli of IgA nephropathy: A new therapeutic opportunity. Oncotarget.

[130] Cokan Vujkovac A., Novaković S., Vujkovac B., Števanec M., Škerl P., Šabovič M. (2020). Aging in fabry disease: Role of telomere length, telomerase activity, and kidney disease. Nephron.

[131] Kooman J.P., Stenvinkel P., Shiels P.G. (2020). Fabry disease: A new model of premature ageing?. Nephron.

[132] Bergmann C., Guay-Woodford L.M., Harris P.C., Horie S., Peters D.J.M., Torres V.E. (2018). Polycystic kidney disease. Nat. Rev. Dis. Primer.

[133] Lu D., Rauhauser A., Li B., Ren C., McEnery K., Zhu J., Chaki M., Vadnagara K., Elhadi S., Jetten A.M. (2016). Loss of Glis2/NPHP7 causes kidney epithelial cell senescence and suppresses cyst growth in the Kif3a mouse model of cystic kidney disease. Kidney Int..

[134] Jin H., Zhang Y., Liu D., Wang S.S., Ding Q., Rastogi P., Purvis M., Wang A., Elhadi S., Ren C. (2020). Innate immune signaling contributes to tubular cell senescence in the Glis2 knockout mouse model of nephronophthisis. Am. J. Pathol..

[135] Larsson S.C., Bäck M., Rees J.M.B., Mason A.M., Burgess S. (2020). Body mass index and body composition in relation to 14 cardiovascular conditions in UK Biobank: A Mendelian randomization study. Eur. Heart J..

[136] Blüher M. (2019). Obesity: Global epidemiology and pathogenesis. Nat. Rev. Endocrinol..

[137] Schafer M.J., Miller J.D., LeBrasseur N.K. (2017). Cellular senescence: Implications for metabolic disease. Mol. Cell. Endocrinol..

[138] Liu Z., Wu K.K.L., Jiang X., Xu A., Cheng K.K.Y. (2020). The role of adipose tissue senescence in obesity- and ageing-related metabolic disorders. Clin. Sci..

[139] Ebert T., Roth I., Richter J., Tönjes A., Kralisch S., Lossner U., Kratzsch J., Blüher M., Stumvoll M., Fasshauer M. (2014). Different associations of adipokines in lean and healthy adults. Horm. Metab. Res..

[140] Palmer A.K., Xu M., Zhu Y., Pirtskhalava T., Weivoda M.M., Hachfeld C.M., Prata L.G., van Dijk T.H., Verkade E., Casaclang-Verzosa G. (2019). Targeting senescent cells alleviates obesity-induced metabolic dysfunction. Aging Cell.

[141] Carracedo M., Witasp A., Qureshi A.R., Laguna-Fernandez A., Brismar T., Stenvinkel P., Bäck M. (2019). Chemerin inhibits vascular calcification through ChemR23 and is associated with lower coronary calcium in chronic kidney disease. J. Intern. Med..

[142] Melk A., Schmidt B.M.W., Vongwiwatana A., Rayner D.C., Halloran P.F. (2005). Increased expression of senescence-associated cell cycle inhibitor p16INK4a in deteriorating renal transplants and diseased native kidney. Am. J. Transplant..

[143] Baisantry A., Berkenkamp B., Rong S., Bhayadia R., Sörensen-Zender I., Schmitt R., Melk A. (2019). Time-dependent p53 inhibition determines senescence attenuation and long-term outcome after renal ischemia-reperfusion. Am. J. Physiol. Ren. Physiol..

[144] Childs B.G., Durik M., Baker D.J., van Deursen J.M. (2015). Cellular senescence in aging and age-related disease: From mechanisms to therapy. Nat. Med..

[145] Luttropp K., Nordfors L., McGuinness D., Wennberg L., Curley H., Quasim T., Genberg H., Sandberg J., Sönnerborg I., Schalling M. (2016). Increased telomere attrition after renal transplantation—Impact of antimetabolite therapy. Transplant. Direct.

[146] Toppe C., Möllsten A., Waernbaum I., Schön S., Gudbjörnsdottir S., Landin-Olsson M., Dahlquist G. (2019). Decreasing cumulative incidence of end-stage renal disease in young patients with type 1 diabetes in Sweden: A 38-year prospective nationwide study. Diabetes Care.

[147] Helve J., Sund R., Arffman M., Harjutsalo V., Groop P.-H., Grönhagen-Riska C., Finne P. (2018). Incidence of end-stage renal disease in patients with type 1 diabetes. Diabetes Care.

[148] Thomas B., Matsushita K., Abate K.H., Al-Aly Z., Ärnlöv J., Asayama K., Atkins R., Badawi A., Ballew S.H., Banerjee A. (2017). Global cardiovascular and renal outcomes of reduced GFR. J. Am. Soc. Nephrol..

[149] Wardyn J.D., Ponsford A.H., Sanderson C.M. (2015). Dissecting molecular cross-talk between Nrf2 and NF-κB response pathways. Biochem. Soc. Trans..

[150] Abreu C.C., Cardozo L.F.M.F., Stockler-Pinto M.B., Esgalhado M., Barboza J.E., Frauches R., Mafra D. (2017). Does resistance exercise performed during dialysis modulate Nrf2 and NF-κB in patients with chronic kidney disease?. Life Sci..

[151] Vega M.R., de la Chapman E., Zhang D.D. (2018). NRF2 and the hallmarks of cancer. Cancer Cell.

[152] Qiao X., Rao P., Zhang Y., Liu L., Pang M., Wang H., Hu M., Tian X., Zhang J., Zhao Y. (2018). Redirecting TGF-β signaling through the β-Catenin/Foxo complex prevents kidney fibrosis. J. Am. Soc. Nephrol..

[153] Zhou X., Wang X. (2015). Klotho: A novel biomarker for cancer. J. Cancer Res. Clin. Oncol..

[154] Dote-Montero M., Amaro-Gahete F.J., De-la-O A., Jurado-Fasoli L., Gutierrez A., Castillo M.J. (2019). Study of the association of DHEAS, testosterone and cortisol with S-Klotho plasma levels in healthy sedentary middle-aged adults. Exp. Gerontol..

[155] Pedersen L., Christensen L.L., Pedersen S.M., Andersen M. (2017). Reduction of calprotectin and phosphate during testosterone therapy in aging men: A randomized controlled trial. J. Endocrinol. Investig..

[156] Neyra J.A., Hu M.C. (2017). Potential application of klotho in human chronic kidney disease. Bone.

[157] Gregg L.P., Hedayati S.S. (2018). Management of traditional cardiovascular risk factors in CKD: What are the data?. Am. J. Kidney Dis..

